# Soybean Hydrophobic Protein is Present in a Matrix Secreted by the Endocarp Epidermis during Seed Development

**DOI:** 10.1038/srep15074

**Published:** 2015-10-12

**Authors:** Daryl E. Enstone, Carol A. Peterson, Mark Gijzen

**Affiliations:** 1 Department of Biology, University of Waterloo, Waterloo, N2L 3G1, Canada; 2 Agriculture and Agri-Food Canada, London, N5V 4T3, Canada

## Abstract

Hydrophobic protein from soybean (HPS) is present in soybean dust and is an allergen (Gly m 1) that causes asthma in allergic individuals. Past studies have shown that HPS occurs on the seed surface. To determine the microscopic localization of HPS during seed development, monoclonal antibodies to HPS were used to visualize the protein by fluorescence and transmission electron microscopy. Seed coat and endocarp sections were also examined for pectin, cellulose, callose, starch, and protein by histochemical staining. HPS is present in the endocarp epidermal cells at 18 to 28 days post anthesis. At later stages of seed development, HPS occurs in extracellular secretions that accumulate unevenly on the endocarp epidermis and seed surface. HPS is synthesized by the endocarp epidermis and deposited on the seed surface as part of a heterogeneous matrix.

Soybean dust is released when seeds are harvested or handled. Epidemic asthma caused the by the large-scale release of soybean dust over cities is well documented^1^,^2^,^3^,^4^. The main allergen responsible for soybean dust-induced asthma is named Gly m 1 and corresponds to the hydrophobic protein from soybean (HPS)^1^. The installation of air handling and filtration equipment is an effective precaution; nonetheless, airborne soybean dust is present in workplaces where soybeans are handled, in port cities where seeds are transferred, and in regions where the crop is grown and harvested^2^,^5^,^6^,^7^,^8^.

Prior to the identification of HPS as an inhalant allergen, the protein was isolated and studied because it has the extraordinary property of spontaneously crystallizing out of solution^9^. Crystals of HPS will precipitate from crude extracts of soybean seeds or hulls, demonstrating the strong self-associative properties of the protein^9^,^10^. HPS belongs to the prolamin super-family of plant proteins and is most closely related to the lipid transfer proteins. HPS is an 8.3 kD alpha-helical bundle with eight cysteine residues that form disulfide bridges^11^,^12^.

The function of HPS is not known for certain but there is evidence indicating that the protein influences the lustre or glossiness of soybean seeds, possibly by mediating the adherence of endocarp secretions or cell fragments to the seed surface^13^,^14^. The present study was undertaken to determine the pattern of HPS secretion and deposition during seed development. Monoclonal antibodies to HPS were used to visualize sites of HPS accumulation by fluorescence and transmission electron microscopy.

## Results

### Adherence of the endocarp epidermis to the seed occurs during the late stages of seed development

**Figure 1:**
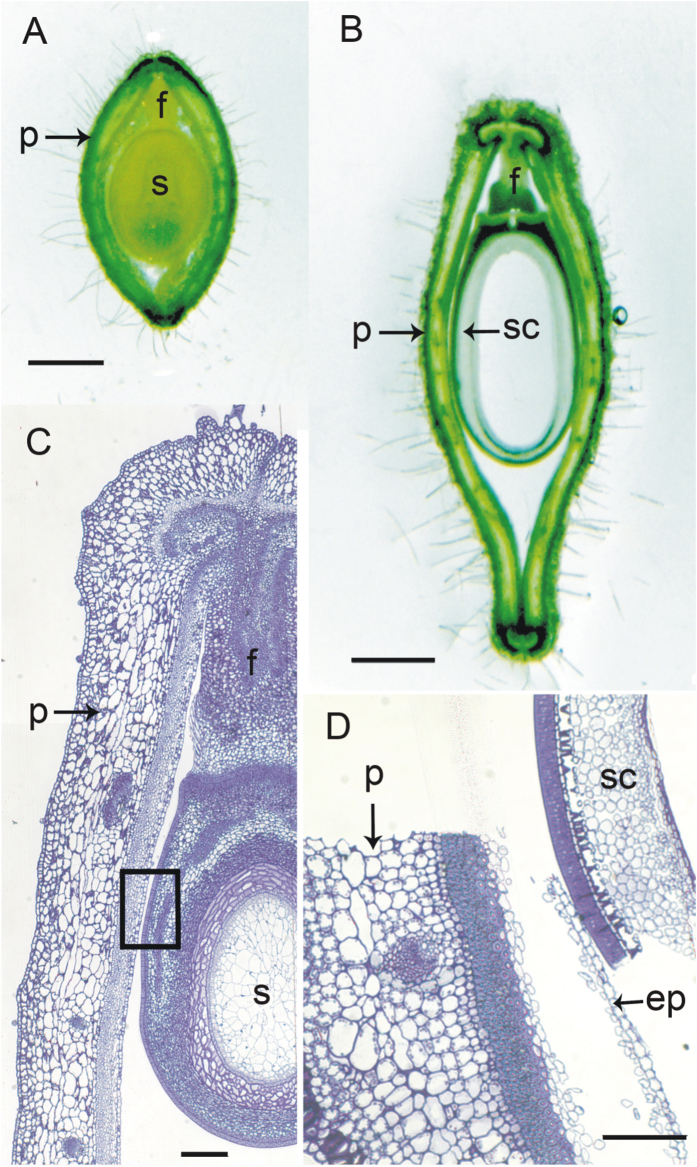
Bright field micrographs of fresh (A,B) and embedded (C,D) sections of soybean pods containing developing seeds. (**A**) 15 dpa fresh cross-section showing the funiculus attachment and the position of the endocarp parenchyma around the developing seed. Bar = 1 mm. (**B**) 25 dpa fresh cross-section; the embryo is missing from the ovule, leaving the only seed coat attached to the pod by the funiculus. Bar = 1 mm. (**C**) 13 dpa thin (1 μm) embedded cross-section showing pod and ovule stained with Toluidine blue O. The box represents an approximate, typical location of a block face within the tissue box trimmed for sectioning. Bar = 20 μm. (**D**) 26 dpa thin (1 μm) embedded longitudinal section of pod endocarp and seed coat, showing extreme example of the displacement of the seed coat from the pod that can occur during processing. Note that the endocarp layer is torn through the parenchyma layer, separating the epidermis from the remainder of the endocarp cells. Bar = 20 μm. ep, endocarp endodermis, f, funiculus; p, pod wall; s, seed; sc, seed coat.

**Figure 2:**
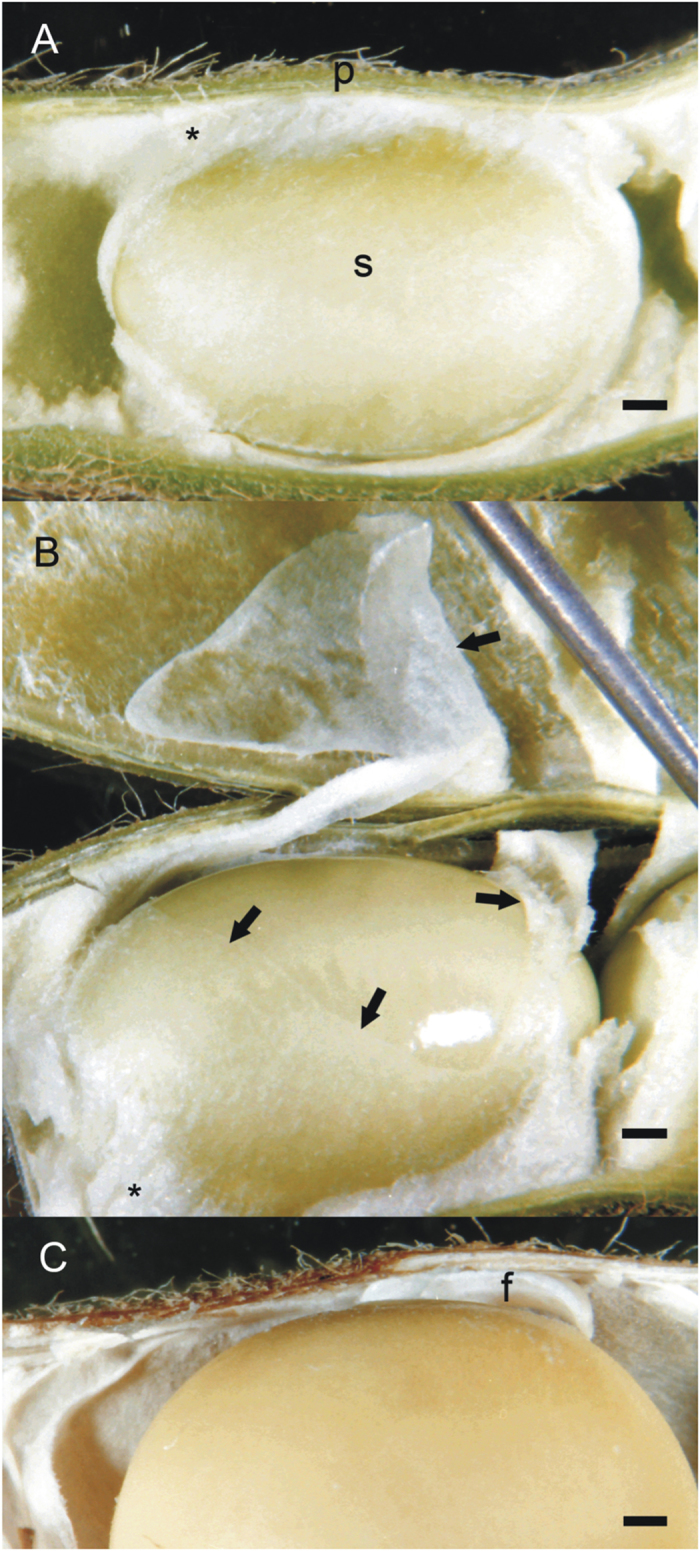
Whole mounts of soybean pods dissected to show the developing seed. Bars = 1 mm (**A**) 25 dpa pod and seed; note the close association of the endocarp and seed. (**B**) 30 dpa pod and seed; part of the endocarp has torn away from the seed and remains partially attached to the inner pod wall. (**C**) 65 dpa pod and seed; the pod and seed are drying and the seed has naturally shrunk away from the pod, pulling the endocarp epidermal layer with it leaving a dull bloom on the seed surface. f, funiculus; p, pod wall; s, seed; asterisk (*), endocarp tissue; arrows, torn endocarp epidermis and parenchyma .

Sections (Fig. 1) and whole mounts (Fig. 2) of soybean seeds within pods are shown to illustrate the organization of reproductive tissues during development and at maturity. Careful dissection of the pod wall away from the developing seeds indicates the relationship between the endocarp and the seed, as shown in Fig. 2. The endocarp epidermis remains in close contact with the seed coat surface in an undisturbed state. The endocarp parenchyma tissue has large air spaces and occludes most of the space around the developing seed (Fig. 2A). As the seed enlarges, this tissue is compressed; however, the endocarp epidermis retains its continuity. The endocarp epidermis of the developing seed does not adhere to the seed coat surface, and dissecting it away exposes the shiny coat (Fig. 2B). In the older, drying seeds, however, the epidermis or portions of it adheres to the seed surface, giving it a dull appearance while the remainder of the pod browns and dries, and pulls away from the seed (Fig. 2C).

### Time course of HPS synthesis and deposition is coordinated with seed development

Multiple samples were collected over time to determine the course of HPS synthesis and deposition. For the interpretation of results, these were grouped into five stages as summarized in Table 1. The stages range from the production of extracellular material on the surface of the endocarp epidermis but no HPS signal (Stage 0), through cellular labelling of HPS and the initial stages of extracellular HPS excretion (Stage 1), to extracellular signal only (Stage 2 onward), and the adherence of both labelled deposits and endocarp cells on the seed coat surface (Stages 3 and 4). Detailed observations from each stage are provided below.

### Stage 0

**Figure 3:**
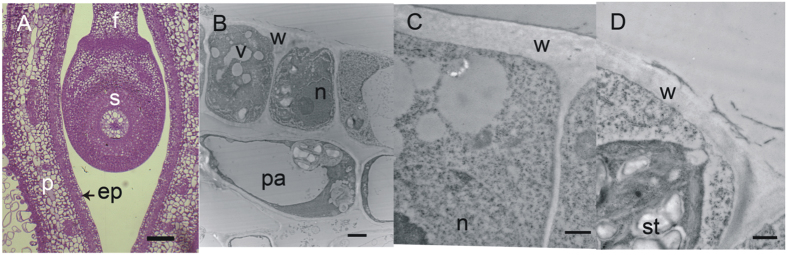
Samples from 10 dpa soybean pods and seeds are negative for the presence of HPS. (**A**) Bright field micrograph of transverse section through pod and developing seed, stained with Toluidine blue O. This is the typical orientation for sample sectioning. Bar = 20 μm. (**B**) Transmission electron micrograph of endocarp epidermis, showing a portion of the outer tangential wall and radial wall, after probing with monoclonal antibody to HPS and immunogold labelling. Bar = 2 μm. (**C**) Transmission electron micrograph of the palisade layer. The cytoplasm is dense with organelles and the vacuoles are still small and not yet coalesced into a single central vacuole. Bar = 500 nm. (**D**) Higher magnification view of endocarp epidermis after probing with monoclonal antibody to HPS and immunogold labelling, showing unlabelled deposits. Bar = 500 nm. ep, endocarp epidermis; f, funiculus; n, nucleus; p, pod wall; pa, endocarp parenchyma; s, seed; st, starch grain; v, vacuole; w, cell wall.

These samples were small enough to cross-section either entirely (10 days post anthesis (dpa), Fig. 3A) or in a bisection (12 to 13 dpa, Fig. 1C). Thus, long stretches of pod and seed coat tissue could be observed in single sections with the light and epi-fluorescence microscope, and with the transmission electron microscope (TEM). The cells of the pod and seed coat (Fig. 3B–D) were densely cytoplasmic with many organelles present. Small amounts of surface material were associated with the endocarp epidermis, indicating that secretions begin prior to 10 dpa. However, the presence of HPS was never detected either by immunofluorescence (not shown) or by immunogold methods (Fig. 3D). The surface of the developing seed coat palisade layer was covered with a conspicuous cuticle (Fig. 3C); however, that of the endocarp epidermis was faint and not always visible in the TEM (Fig. 3B,D).

### Stage 1

**Figure 4:**
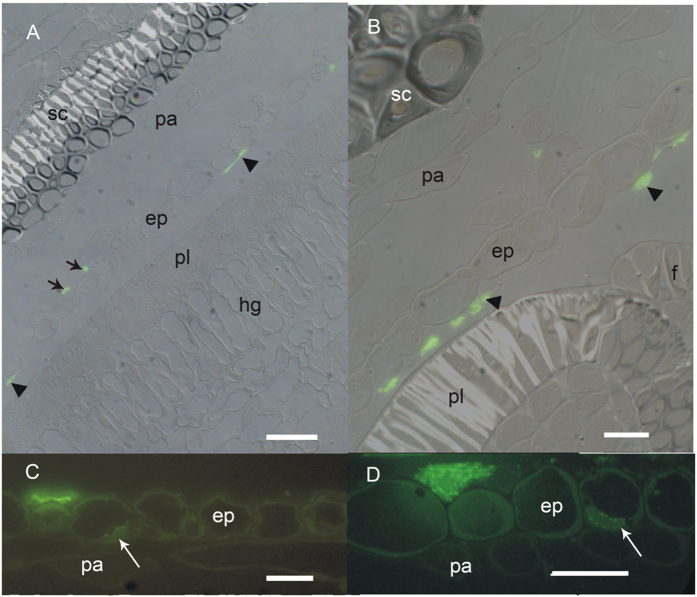
Samples from 18 to 28 dpa soybean pods and seeds test positive for the presence of HPS. All sections shown after probing with monoclonal antibody to HPS and Oregon Green reporter conjugate. (**A**) An18 dpa section with HPS occurring on the endocarp epidermal surface adjacent to the seed coat (arrowheads) and, less commonly, on the interior parenchyma side of the endocarp epidermis (arrows). This figure is an overlay of fluorescence and differential interference contrast images, showing HPS on the surface of the endocarp epidermis. Bar = 50 μm. (**B**) An overlay view of endocarp in the hilum region of an ovule at 21 dpa. The HPS deposit (arrowheads) extends beyond the region of the seed coat and is also adjacent to the funiculus. Bar = 20 μm. (**C**) Detail of the endocarp epidermis at 21 dpa, showing surface deposits of HPS; label is also faintly present in the cytoplasm and in the nucleus (arrow). Bar = 20 μm. (**D**) 28 dpa endocarp epidermal cells showing closer view of HPS occurring within deposits on surface and faint HPS signal in the nucleus (arrow). Note that the HPS signal is not evenly distributed within the deposits. Bar = 50 μm. ep, endocarp epidermis; f, funiculus; hg, hourglass cells; pa, endocarp parenchyma; pl, palisade cells; sc endocarp sclerenchyma.

**Figure 5:**
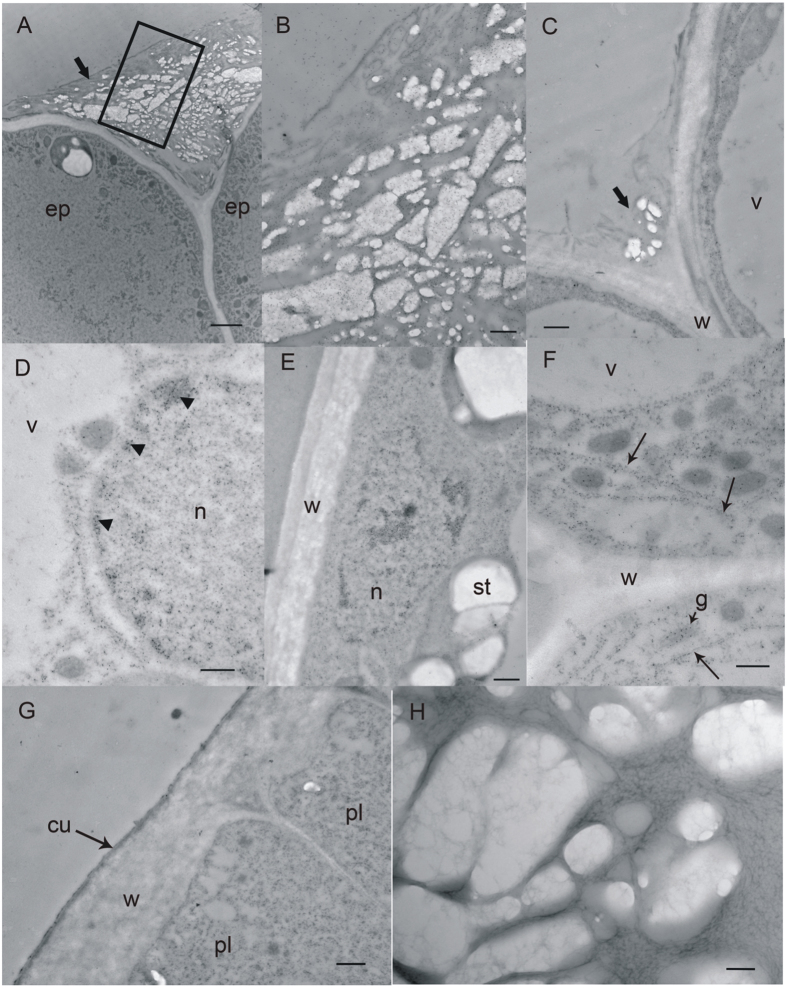
Transmission electron microscopy of endocarp and seed coat showing HPS deposition. Panels (**A**–**G**) show samples labeled with monoclonal antibody to HPS and immunogold; panel (**H**) stained with uranyl acetate and lead citrate, but not labeled with immunogold. (**A**) Deposits on surface of 26 dpa endocarp epidermis (bar = 2 μm); box indicates area enlarged in (**B**), showing heterogeneous surface deposits with gold label. (Bar = 500 nm.) (**C**) Endocarp epidermis showing labeled surface deposit. (Bar = 500 nm.) (**D**) Endocarp epidermis with nucleus (n) and vacuole (v), showing gold label localized to dense heterochromatic nuclear regions (arrowheads). (Bar = 500 nm.) (**E**) Endocarp epidermal cell with labeled nucleus. Large, unlabeled starch grains are present. (Bar = 500 nm.) (**F**) Endocarp epidermal cell showing gold label associated with rough endoplasmic reticulum (arrows) and golgi within cytoplasm; cell wall and vacuole also shown, but minimally labeled. Bar = 500 nm. (**G**) Seed coat palisade cells showing dense cytoplasm, electron-dense cuticle on surface and lack of gold label (Bar = 500 nm). (**H**) Detail of endocarp epidermal surface deposits revealing a fine reticulate network of moderately electron-dense material surrounding large electron-lucent bodies. (Bar = 100 nm). cu, cuticle; ep, endocarp epidermis; g, golgi body; hc, heterochromatic regions of nucleus; pl, palisade cells; n, nucleus; st, starch grain; v, vacuole; w, cell wall.

From this sample stage on, the embedded pod specimens were too large to section intact; instead they were subdivided into smaller pieces and mounted and sectioned separately. This stage was characterized by the appearance of HPS in the endocarp, both in deposits on the epidermal surface (Fig. 4A–D, and Fig. 5A,C) and within some of the epidermal cell protoplasts (Fig. 4C,D, and Fig. 5D–F). Occasionally, HPS-labelled material was found in the air spaces of the endocarp parenchyma near the epidermis (Fig. 4A), but not in the parenchyma protoplasts. The younger specimens at this stage (18 to 21 dap) had endocarp epidermal and parenchyma cells with large central vacuoles and peripheral cytoplasm packed with many organelles, and amyloplasts with large starch grains and some grana stacks (Fig. 5A,C–F). The palisade layer had a similar cytoplasmic appearance, but immunogold label was not present in the palisade protoplasts (Fig. 5G). In older samples of this stage the peripheral cytoplasm became slightly thinner and less packed with organelles. An amorphous and heterogeneous material was present in greater amounts on the surface of the endocarp epidermis than seen in Stage 0 specimens. The material was present on the inner pod surface from the funiculus (Fig. 4B) to the ventral suture. It was not uniformly distributed but in sectional view appeared as clumps (Fig. 4). These clumps were larger and more closely spaced in the older samples. The Oregon green-labelled material appeared to be a fibrillar structure (Fig. 4D). Cytosolic label, where present, was faint (Fig. 4D), but slightly brighter spots were frequently visible in association with the nucleus (Fig. 4C,D). Examination of cellular fine structure with immunogold confirmed that the endocarp epidermis cytoplasm did contain label which was often associated with rough endoplasmic reticulum and Golgi, as well as being in the cytosol (Fig. 5F). Label was variably present in the vacuole; usually it appeared where the tonoplast was disrupted, and the gold was associated with darker material in the vacuolar region. Label was also present in the nucleus, on average at a low level, but concentrated in the darker regions of heterochromatin (Fig. 5D,E). Within the surface deposits, gold label was associated with an electron-dense material that formed a network around domains of electron-lucent material (Fig. 5A,B). This deposition pattern was very similar to networks of more electron-dense material in sections stained with uranyl acetate and lead citrate (Fig. 5H). The electron-lucent material did not infiltrate well and the plastic frequently tore in these spots, resulting in enlarged, empty oval spaces. Cytoplasmic label was not seen within the endocarp parenchyma or sclerenchyma, or within the cells of the seed coat.

### Stage 2

From 30 dpa and onward, the HPS signal became exclusively extracellular and confined to the secreted deposits. The vast majority of this material was between the endocarp epidermis and the seed coat surface. HPS-labelled deposits were seen within the endocarp parenchyma air spaces, usually immediately adjacent to the endocarp epidermis, but occasionally deeper within the parenchyma, toward the sclerenchyma layers. Handling stresses often caused separation between the endocarp epidermis and the ovule surface (Fig. 1D). In these cases, the secreted deposits remained with the endocarp. The parenchyma layer also tore, resulting in the innermost part of the endocarp appearing separate from the remainder of the pod.

### Stage 3

**Figure 6:**
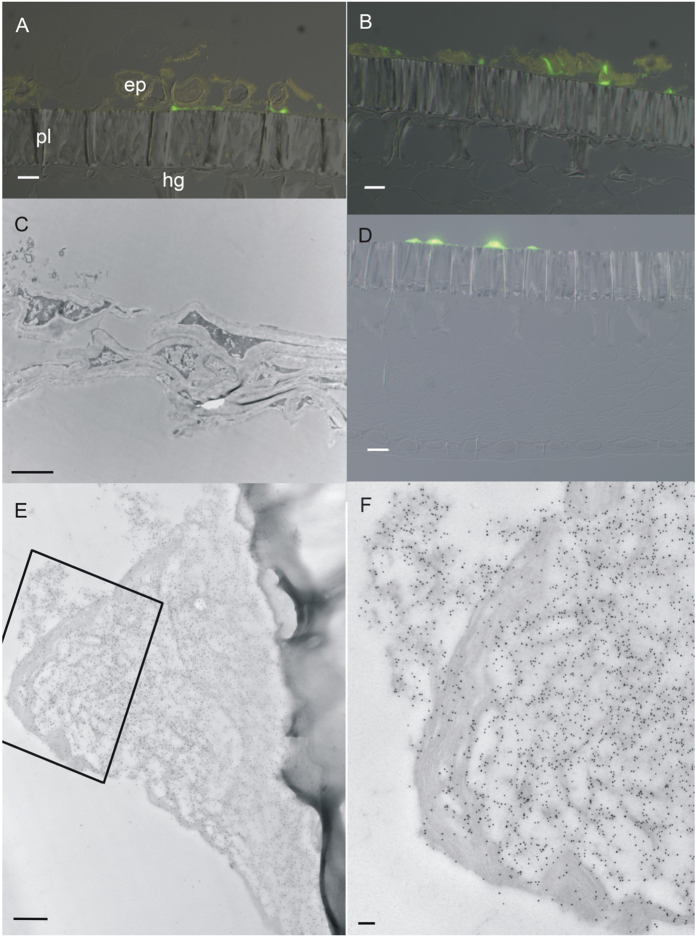
Deposition of HPS during late stages of seed development. Panels (**A**–**C**) from 55 to 60 dpa, panels (**D**–**F**) from fully mature, dry seeds. (**A**) Overlay of fluorescence and differential interference contrast images of endocarp epidermis after probing with monoclonal antibody to HPS and Oregon Green reporter conjugate, showing endocarp epidermal and parenchyma cells adhering to the palisade layer of the seed coat. Labeled material is located between the endocarp and seed coat cells. Bar = 50 μm. (**B**) An overlay similar to (**A**) in an adjacent region of the section showing twisting and distortion of endocarp layers. Bar = 50 μm. (**C**) Transmission electron micrographs of endocarp cells showing their flattened and distorted shape at this stage of development, for comparison with Fig. 6B. Bar = 2 μm. (**D**) Overlay of fluorescence and differential interference contrast images of seed coat after probing with monoclonal antibody to HPS and Oregon Green reporter conjugate, showing HPS deposits on the seed coat surface but not in other areas of the section. Bar = 50 μm. (**E**,**F**) Transmission electron micrographs of samples labeled with monoclonal antibody to HPS and immunogold, showing deposit on the seed coat surface. Boxed area in (**E**) corresponds to enlarged area shown in (**F**). Note the heterogeneous nature of the deposits with a reticulate network surrounding electron-lucent regions and additional areas of laminar material. Gold label is associated with the reticulate network but not with the lucent embedded material. Bars = 500 nm (**E**) and 100 nm (**F**); ep, endocarp epidermis; pl, palisade layer; hourglass layer.

At 50 dpa, HPS-labelled surface material was present on both the endocarp epidermal surface and the seed coat surface. The endocarp epidermis was almost always separated from both pod and seed in 50 dpa samples. In the samples from 55 dpa and older, endocarp cells were also present on the seed coat, along with the labelled surface material (Fig. 6A,B). In these latter samples, the pod wall was dissected away from the seed and only the coat was selected for processing, so that a separation was forced at the time of sampling. Some of the endocarp tissue that remained with the seed coat was embedded in the plastic, but detached from and adjacent to the coat surface (Fig. 6C), indicating that it had been pulled away from the palisade surface during the processing. Alternatively this tissue may have been anchored by attachment to other endocarp cells that adhered to the coat surface but were not in the plane of the section. These pod cells were often highly distorted, twisted and sometimes collapsed with portions of the same cell or adjacent cell walls intruding into the lumena. Endocarp epidermis and some of the adjacent parenchyma layers adhered to the coat surface. HPS label occurred in patches of deposits on the coat surface, between the adhering endocarp cells and the coat surface, and on portions of the surface of cells that were torn away from the surface. The labelled deposits did not extend all around the endocarp cells and some cells (probably parenchyma) had no deposits on the surface (Fig. 6A,B).

### Stage 4

Mature, fully dry seed coats had HPS label in patches on the seed coat surface (Fig. 6D). As in the developing coats, the surface deposits were heterogeneous with gold-labeled, darker material forming a reticulated network around highly lucent amorphous material (Fig. 6E,F). Frequently, a highly laminar material was included in the more protruding deposits. Other portions of the coat surface simply had a very thin layer of label on the seed coat surface, interspersed with regions that had no label. Individual endocarp cells were not identifiable in these samples.

### Histochemical Staining

**Figure 7:**
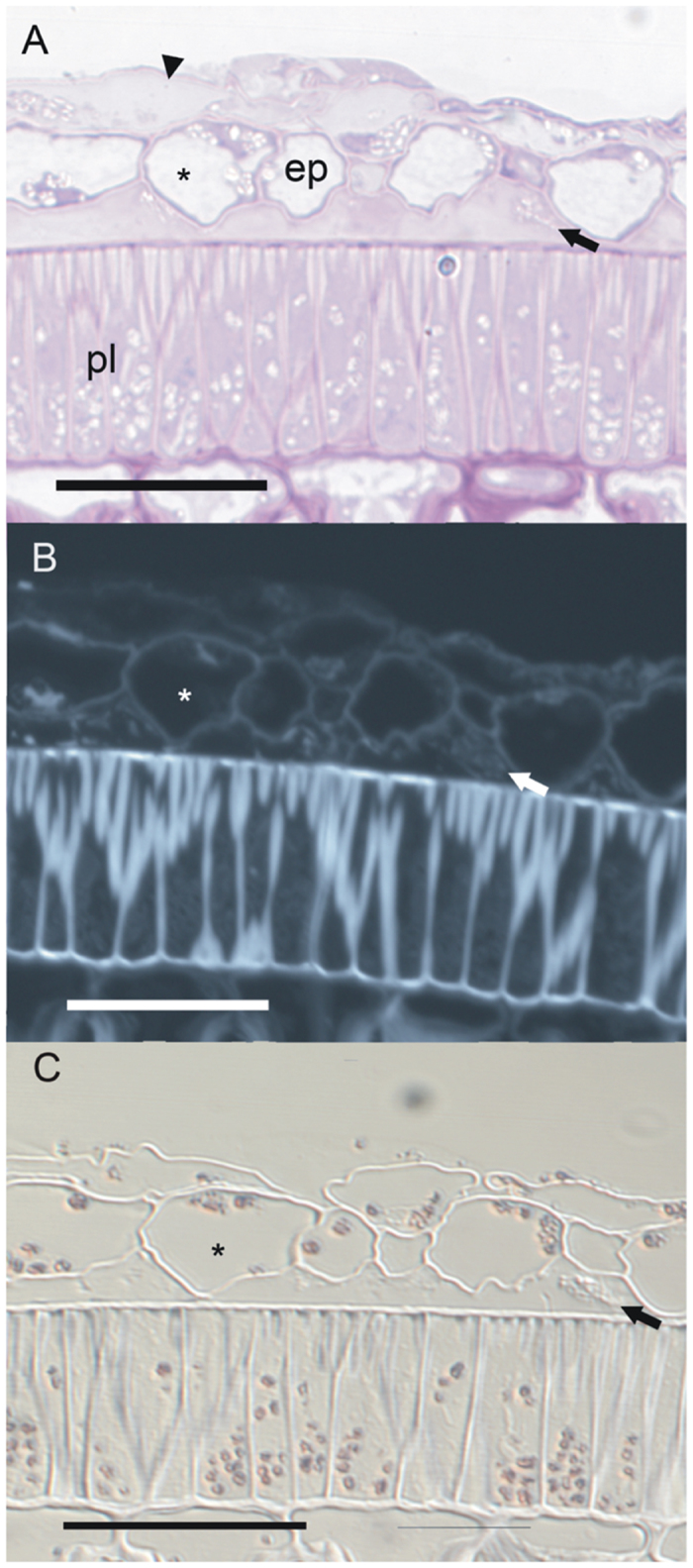
Histochemical staining of embedded thin sections of 30 to 48 dpa soybean seedcoat and endocarp tissues. The asterisk indicates the same endocarp epidermal cell and the arrow indicates the same surface deposit in each figure. Bars = 50 μm. (**A**) Bright field view of Toluidine blue O stained section; pink colour indicates presence of pectin. Note that the surface deposits contain pink-coloured areas and unstained areas. Arrowhead indicates collapsed endocarp parenchyma. ep, endocarp epidermis; pl, palisade layer. (**B**) Fluorescence image with UV excitation of cellufluor-stained section; blue-white fluorescence indicates cellulose or other β-1,4-glucose-containing polymer. The surface deposits contain some cellufluor fluorescence indicating that there may be cellulose within the deposits, but as with the pectin deposits (7A), the staining is not even within the deposit. (**C**) Bright field image of I_2_KI-stained section showing brown starch grains in the palisade and hourglass layers of the seed coat, and in the epidermis and parenchyma of the endocarp, but no staining of the surface deposits. The deposits have a heterogeneous appearance with DIC illumination.

LR White-embedded sections of both mature (Stage 4) and immature (Stage 2) coat with associated endocarp epidermis were histochemically stained to assess carbohydrate composition (Table 2). The matrix material that contains HPS signal contained pectic material as indicated by pink-purple Toluidine blue O staining (Fig. 7A). Within the deposits there were also unstained spots, indicating the heterogeneous nature of the material and suggesting components that are pectinaceous and non-pectinaceous. The cellufluor staining for cellulose was particularly bright in the seed coat palisade and hourglass cell walls whereas the walls of the endocarp epidermis and parenchyma were stained far less (Fig. 7B). At stage 2, the palisade and particularly the hourglass cells were forming thick cellulosic secondary walls that had not yet lignified (no blue-green stain with Toluidine blue O to indicate lignin), and this is why these walls appeared bright in comparison to the thin, primary walls of the endocarp cells. In the Stage 2 endocarp epidermis there was cellufluor - stained material in some of the cells and surface deposits (Fig. 7B); however, surface deposit staining in mature material was extremely faint. This decrease in fluorescence may indicate an infiltration of tannins (that have a quenching effect) from dying endocarp cells, rather than a loss of cellufluor-stained material. Aniline blue stained the surface deposits of both Stage 2 and Stage 4 specimens very light blue but no fluorescence under violet excitation was present indicating a lack of callose (not shown). The I_2_KI stain revealed prominent starch grains in the endocarp epidermis and parenchyma as well as the seed palisade and hourglass layers (Fig. 7C). Features similar to these intracellular starch grains appear in the extracellular surface deposits (Fig. 7A), but the extracellular material did not stain with I_2_KI (Fig. 7C). In short, the electron-lucent material embedded within this matrix does not contain callose or starch, according to the staining analysis. Small amounts of pectins, plus cellulose or another, similar, polymer appear to be present.

## Discussion

Past studies have shown that HPS is an unusual and allergenic protein that is localized to the seed surface^1^,^13^,^14^,^15^. The amount of HPS present on the seed varies among soybean cultivars and is associated with dull seed lustre^14^. Most commercially grown soybeans are classified as having either a dull or shiny seed lustre. We have now determined the microscopic localization of HPS and describe how the protein is embedded in a heterogeneous matrix that is secreted by the endocarp epidermal cell layer during the course of seed development.

During the early stages of seed development, from 10 to 13 dpa, the endocarp epidermis appeared to commence secretion of material but HPS was not detected. At this stage, the epidermal endocarp is composed of regular, cube-shaped cells^16^. However, the endocarp cuticle, which lines the interior of the pod, is beginning to disintegrate and separate from the cell wall.

At 18 to 28 dpa, HPS is detectable in endocarp epidermal cells and in secretions from the endocarp epidermis along the entire interior of the pod. Within endocarp epidermal cells HPS is detected in the rough endoplasmic reticulum, golgi, and cytosol, as might be expected for a secreted protein. Surprisingly HPS was also detected in heterochromatic regions of the nucleus, but we do not have an explanation for this observation. Regardless, the strongest signals for HPS were found in extracellular secretions that occurred in clumps, unevenly distributed along the surface of the epidermis. Close examination of the secretions by transmission electron microscopy indicate that seed surface deposits with HPS are formed by the endocarp epidermis along the entire interior of the pod. We also noted that secretion of HPS does not start simultaneously in all cells, as the deposits tend to be interspersed, especially in younger samples compared to older samples. Fluorescence and DIC overlays indicate that not all surface deposits contain HPS. In 26 dpa specimens, some deposits are labelled, but others are not. Analysis by immunogold labelling indicates that only deposits containing large oval electron-lucent regions have large amounts of HPS-label. Other regions contain deposits consisting of a laminar-like structure that do not have HPS-label or have very low levels of label.

## Conclusions

Results from this study show that HPS synthesis and secretion by the endocarp epidermis begins between 13 and 18 dpa. HPS is one component present in a heterogeneous secreted matix that is deposited along the whole of the pod endocarp from the funiculus to the ventral suture. The function of these endocarp secretions is not known. It is possible that the deposits assist during the expansion of the seed by allowing the seed coat surface to slide more easily over the surface of the pod endocarp. This idea is based on the observation that the alignment between the seed coat and epidermis of the pod endocarp shifts during seed growth and development. There is also a likely role for the deposits in mediating the attachment of remnants of the endocarp epidermis to the seed surface at seed maturity. Certainly, previous work has established a clear association between the amount of HPS and the seed surface lustre, with greater amounts of HPS occurring on dull-seeded cultivars^13^. Furthermore, there is evidence indicating that the *HPS* genetic locus controls the amount of HPS protein produced and that it corresponds to the bloom (*B*) gene that conditions dull seeded phenotypes^14^,^17^. The present study demonstrates that HPS is embedded in a matrix of secreted materials. This raises new questions, such as what are the other components of the secreted matrix, and are they universally produced by soybean cultivars, regardless of the amount of HPS protein produced? The characterization of these deposits may offer opportunities for selection and improving seed quality in soybean.

## Methods

### Plant production and pod collection

Soybean seeds (*Glycine max* [L.] Merr., cv Harosoy 63) were obtained from the collection at Agriculture and Agri-Food Canada. The cultivar Harosoy 63 was selected because it is known to produce seeds with abundant HPS^13^. Plants were grown on field plots outdoors, on the AAFC experimental farm in London ON. No specific permission was required for this location or activity. The experiments did not involve the use of transgenic plants. The field use did not involve endangered or protected species. Flowers were tagged on the day of opening and subsequently the developing pods were collected, either from the same inflorescences or those of the same age based on location on the plant and stage of pod development.

### Sample preparation for immunolocalization of HPS

Collected pods were transported to the laboratory on ice for immediate sample processing. Transverse sections 1 mm in thickness were cut through the pod and developing seeds (Fig. 1A,B). In the youngest samples taken from10 to 12 days post anthesis (dpa), the cotyledons remained in place during this step (Fig. 1A). In older samples, the cotyledons were carefully removed from the seed coat, so that only seed coat and pod wall were fixed (Fig. 1B). An ideal section consisted of the pod wall with the seed coat held within it by the funiculus. These sections were immediately transferred to fixative consisting of 0.1% glutaraldehyde (Sigma-Aldrich, St. Louis MO) and 2% paraformaldehyde (Electron Microscopy Supply (EMS), Hatfield PA) in 25 mM phosphate buffer, pH 7.2. Initial fixation was carried out for 1 h at room temperature on a rotator. After transfer to fresh fixative, sample treatment continued overnight at 4 °C. For samples destined for electron microscopy, post-fixation with 2% OsO4 (EMS) for 4 h was included to increase tissue contrast. All tissues were dehydrated through an ethanol series starting with 30 min in 25% ethanol and followed by 1 h steps in each of 55%, 70%, 95%, and 2 × 100% solutions. All stages were carried out at room temperature on a rotator. This protocol ensures adequate infiltration of resin into the mature layers of endocarp and seed coat, as advised by Dr. Fengshan Ma (personal communication). Samples progressed through a series of 1:3, 1:1, 3:1, 95%, and 4 × 100% LR White (hard grade, Canemco Inc, Canton de Gore, Lakefield, QC) for a minimum of 2 h each on a rotator at room temperature. When overnight breaks were necessary, the samples remained in the current solution at room temperature and rotated. For embedding, the sections were gently transferred to fresh LR White in a flat mold, sealed, and fixed at 55 °C for approximately 20 h or until polymerized (up to 24 h).

Blocks containing sections were cut from the resin and mounted on acrylic stubs (Ted Pella, Inc, Redding CA). In most cases, the sections were mounted transversely (i.e., with the cut tissue edge up, to produce transverse pod sections) as shown in Fig. 1C; however, in some cases they were mounted so as to provide longitudinal sections of pods and seed coats (Fig. 1D). Sections for immunolabelling were cut using glass knives with an ultramicrotome (Leica Reichart-Jung Ultracut E, Vienna, Austria). Thick sections of 0.5 to 1.0 μm for immunofluorescence labelling were mounted on coated slides (Gold Seal Ultrastick, EMS). Ultrathin sections (silver to gold) for immunogold labelling were mounted on formvar-coated nickel grids (EMS).

### Immunocytochemistry

Two monoclonal antibodies against HPS, 6-G-1 and 1-G-10, produced in mouse cells (Alk-Abelló, Spain) were used for protein localization. For immunofluorescence labelling, sections were cut, labelled and observed on the same day. The secondary antibody was goat anti-mouse IgG labelled with Oregon Green 488 (Molecular Probes/Thermo Fisher, catalog number O6380).Sections were examined using a Zeiss Axiophot epifluorescence microscope fitted with a blue filter set (excitation BP 450–490, chromatic beam splitter FT 510, barrier LP 520). Successful labeling of HPS appeared as regions of bright green fluorescence emission associated with identifiable plant structures. Photographs were taken with a digital camera (Retiga, QImaging, Surry BC) controlled by Openlab software (Improvision Inc., Waltham, MA USA).

The immunolabeling protocol was similar for both immunofluorescence and immunogold samples and was kindly provided by Dr. John Greenwood. Immunofluoresce sections were stained on-slide in a humid chamber (lidded petri dish containing wetted filter paper and shielded with foil). Nickel grids were transferred between droplets of solutions on a length of Parafilm; the droplets were covered by a culture dish lid to prevent evaporation. Rinse solutions were purified water and phosphate buffered saline (PBS), and the blocking agent was a solution of PBS with the saline at 3x usual concentration, 0.5% Tween 20, glycine 0.05%, and bovine serum albumin (BSA) 0.5% (PB3xSTGBSA). To immunolabel, the samples were exposed to 0.1N HCl for 5 min, rinsed 3x with water, treated with PB3xSTGBSA for 10 min, then exposed to primary monoclonal antibody for 30 min. This was followed by 3 changes of PB3xSTGBSA (3 min each) and labeling with secondary antibody or control solution for 30 min. Finally, the sections were rinsed with 3 changes PB3xSTGBSA (3 min each), 3 changes PBS (3 min each) and 3 changes water (3 min each). Immunofluorescence sections were mounted in 50% glycerol in PBS with two drops of FluoroGuard (Bio-Rad) to protect from fluorescence fading.

Ultrathin sections for transmission electron microscopy were cut and labelled on the same day. The immunogold labeling protocol started with 1 h in 12.5% aqueous sodium metaperiodate followed by 3 brief rinses with water. A goat anti-mouse antibody conjugated to 10 nm gold particles (Sigma-Aldrich, cat. G777) was utilized as the secondary label. At the end of the labeling procedure, the immunogold samples were stained with 3% aqueous uranyl acetate for 10 min, followed by 3 rinses with water to enhance contrast within the tissue. All intervening steps in the labeling were identical to those for immunofluorescence. The grids were examined with a transmission electron microscope (Philips CM10) equipped with a digital camera and software (Advanced Microscopy Technologies, Danvers, MA). Successful HPS labelling appeared as small, electron opaque circles measuring approximately 10 nm diameter which were associated with structures within the plant tissue.

Two controls were run in parallel to the antibody treatment. The first control contained no primary monoclonal antibody and no secondary antibody. The second control contained no primary, but did contain secondary antibody. The first controls showed only background tissue autofluorescence (in the case of immunofluorescence) and showed no sign of radio-opaque dots of 10 nm diameter (in the case of immunogold samples). The second control provides a comparison of the effects of the secondary antibody on background fluorescence. This was minimal with no bright spots or patches (in the case of immunofluorescence samples), or only a very small, random scattering of radio-opaque dots in the resin borders outside the tissue equal to the amounts visible over the tissue region (in the case of immunogold samples).

Digital images were handled in software to prepare the illustrations (Adobe Photoshop 3 and Microsoft Office PowerPoint 2003). For most of the immunofluorescence images, the contrast and brightness were adjusted to improve detail.

### Histochemistry

Histochemical tests were performed on Stage 2 and Stage 4 (Table 1) embedded specimens to assess the types and locations of carbohydrates in the seed coat palisade layer, the endocarp epidermis, and in the deposits formed on the seed coat surface. The stains used were: alkaline Toluidine blue O (0.5% Toluidine blue O in 1% sodium borate, aqueous) for pectin and lignin; aniline blue WS (AB, 0.5% aqueous) for callose^18^; cellufluor (0.001% aqueous) for cellulose^19^; and iodine potassium iodide (containing 2% KI aqueous (2 g/100 mL) and 0.2% iodine (as crystals) (0.2 g/100 mL IKI) for starch^18^. Gentle heating was applied to the sections in solution to enhance staining.

## Additional Information

**How to cite this article**: Enstone, D. E. *et al*. Soybean Hydrophobic Protein is Present in a Matrix Secreted by the Endocarp Epidermis during Seed Development. *Sci. Rep*. **5**, 15074; doi: 10.1038/srep15074 (2015).

## Figures and Tables

**Table 1:** Stages of soybean seed development and summary of HPS detection in seed and endocarp.

Stage	Age (dpa)*	Observations
0	10 to 13	Small surface deposits present on endocarp epidermal surface, but no HPS signal within cell protoplasts or in the deposits.
1	18 to 28	Initial appearance of HPS signal within cells of the endocarp epidermis, including nucleus. Extracellular export of HPS-labelled material.
2	30 to 48	HPS signal is solely extracellular from this point on. HPS signal is typically associated with the endocarp surface, where that layer has separated from the seed coat.
3	50 to 60	At 50 dpa, HPS signal is present on the seed coat surface as well as the separated endocarp surface. By 55 to 60 dpa, endocarp epidermis and parenchyma cells are present along with HPS signal on the seed coat surface.
4	Mature	Pods and seed are dry, and the pod is ready to open. The seed coats are dull when the pod wall is dissected away. HPS is present on the seed coat surface together with other materials.

^*^dpa, days post anthesis.

**Table 2:** Summary of histochemical stain results for surface deposits in immature and mature seed coat and endocarp sections.

Stain	Target (colouration)	Result	
		Immature (Stage 2)	Mature (Stage 4)
Toluidine blue O	Pectins (pink-purple)	Positive	Positive
	Lignin (blue-green)	Negative	Negative
Cellufluor	Cellulose (blue-white fluorescence with UV)	Positive	Faint positive
I_2_KI	Starch (brown or black)	Negative	Negative
